# Role of Acyl-CoA Thioesterase 7 in Regulating Fatty Acid Metabolism and Its Contribution to the Onset and Progression of Bovine Clinical Mastitis

**DOI:** 10.3390/ijms252313046

**Published:** 2024-12-04

**Authors:** Bin Zhou, Bohao Zhang, Jiangyuan Han, Junjun Zhang, Jianfu Li, Weitao Dong, Xingxu Zhao, Yong Zhang, Quanwei Zhang

**Affiliations:** 1 College of Life Sciences and Biotechnology, Gansu Agricultural University, Lanzhou 730030, China; zhoub@st.gsau.edu.cn (B.Z.); hanjiangy@gsau.edu.cn (J.H.); jun2272136040@126.com (J.Z.); li18419220108@163.com (J.L.); zhaoxx@gsau.edu.cn (X.Z.); zhychy@126.com (Y.Z.); 2 Gansu Key Laboratory of Animal Generational Physiology and Reproductive Regulation, Lanzhou 730070, China; zhangbhgs@163.com (B.Z.); d.wt2008@163.com (W.D.); 3 College of Veterinary Medicine, Gansu Agricultural University, Lanzhou 730070, China

**Keywords:** clinical mastitis, long-chain fatty acids, ACOT7, bioinformatics

## Abstract

Clinical mastitis (CM) is a prevalent and severe inflammatory disease in dairy cows affecting the mammary glands. Fatty acid (FA) metabolism and associated enzymes are crucial for many physiological and pathological processes in dairy cows. However, the relationships among FA metabolism, FA-associated enzymes, and CM, as well as the mechanisms underlying their interactions, in dairy cows are not fully understood. The aim of this study was to characterize biological process (BP) terms, pathways, and differentially expressed proteins (DEPs) related to FA metabolism from our previous data-independent acquisition proteomic study. Six BPs involving 14 downregulated and 20 upregulated DEPs, and four pathways involving 10 downregulated and 11 upregulated DEPs related to FA synthesis and metabolism were systematically identified. Associated analysis suggested that 12 candidate DEPs obtained from BPs and pathways, especially acyl-CoA thioesterase 7 (ACOT7), regulate long-chain FA (LCFA) elongation and the biosynthesis of unsaturated FAs. Immunohistochemical and immunofluorescence staining results showed that ACOT7 was present mainly in the cytoplasm of mammary epithelial cells. The qRT-PCR and Western blotting results showed that *ACOT7* mRNA and protein levels in the mammary glands of the CM group were significantly upregulated compared to those in the healthy group. This evidence indicates that ACOT7 is positively correlated with CM onset and progression in Holstein cows. These findings offer novel insights into the role of FA metabolism and related enzymes in CM and offer potential targets for the development of therapeutic strategies and biomarkers for the prevention and treatment of CM in dairy cows.

## 1. Introduction

Clinical mastitis (CM), a prevalent and severe disease in dairy cows, is triggered by physical, chemical, and microbial factors that lead to localized inflammation in the bovine mammary glands. The mammary gland, as the organ for milk production, functions normally in lactation, producing various fatty acids (FAs), proteins, and other minor components of milk. FAs are crucial components of milk fat and are a key energy source for mammary epithelial cells (MECs) [1,2]. MECs either take up free FAs from the plasma or synthesize them internally, esterify them into various FAs, and secrete them into milk [2]. The endogenous accumulation or unbalanced production of FAs, occurring both naturally in the mammary gland or upon exogenous microbial infection with Gram-negative bacteria, results in the production of endotoxins, such as lipopolysaccharide (LPS), which translocate into the bloodstream and lead to an inflammatory response [3], particularly in the mammary gland. Consequently, pathogen infections prompt the immune system to initiate inflammatory responses in the mammary gland, resulting in tissue damage and disturbances in FA metabolism [4,5], which manifest as typical CM-associated characteristics of bovine mammary glands and milk. This severely affects milk yield and quality, resulting in decreased milk production, higher veterinary expenses, and prolonged herd management challenges, which collectively have a substantial impact on the economic sustainability of the dairy industry [6,7]. However, the pathological molecular mechanisms associated with FAs during the regulation of CM development in dairy cows are not completely understood.

FAs, including short-chain FAs (SCFAs), long-chain FAs (LCFAs), and medium-chain FAs (MCFAs), depending on their type, intake, or consumption patterns and specific health conditions, can affect multiple aspects of inflammation, such as leukocyte chemotaxis, adhesion molecule expression, and leukocyte–endothelial adhesive interactions [6]. This is especially apparent with chronic inflammatory diseases, such as mastitis, ketoacidosis, and endometritis in mammals [8,9]. For example, SCFAs can modulate the production of inflammatory mediators, such as interferon-gamma (IFN-γ), interleukin (IL)-2, and IL-10 in inflammatory cells [10]. SCFAs are the potential immunomodulatory metabolites in controlling *Staphylococcus-aureus*-mediated mastitis [11]. Further, saturated FAs (SFAs), a subset of LCFAs, are closely associated with chronic inflammation [12]. Elevated SFA levels can activate the Toll-like receptor (TLR) 4 signaling pathway, leading to the increased production of pro-inflammatory cytokines, such as tumor necrosis factor-alpha (TNF-α) and IL-6, thereby promoting the onset of mastitis [13,14]. Levels of these pro-inflammatory cytokines are upregulated during the pathogenesis of inflammation-based mastitis in dairy cows [15]. Moreover, excessive SFA intake can aggravate mastitis in dairy cows, which is dependent on alterations to the fluidity of cell membranes, disruptions in signal transduction, and enhanced lipid synthesis, intensifying the inflammatory response in MECs [16]. Considering the role of fatty acids in promoting the upregulation of inflammatory cytokines, it is plausible to hypothesize that this process may be driven by dysregulation of fatty acid metabolism, thereby contributing to the development of mastitis. Interestingly, owing to the beneficial effects of MCFAs on anti-pathogen activity, immune-improving reactions, inflammation prevention, and the suppression of damage caused by inflammation, they have been used as feed supplements for monogastric animals [17]. However, their application in ruminants remains controversial. Collectively, FA metabolism has crucial roles in various physiological and pathological processes in animals, both as an important source of energy and a key component of cell membranes, while occupying an important position in signal transduction and metabolic regulation.

Enzymes associated with FA metabolism can influence inflammatory responses via different pathways. Fatty acid synthase (FASN), a key metabolic enzyme, is significantly correlated with the expression of inflammatory cytokines and enzymes in bovine MECs [7]. Moreover, acyl-CoA thioesterases (ACOTs), comprising a key class of lipases responsible for the β-oxidation of LCFAs [18,19], can affect various inflammatory signaling pathways via LCFA accumulation [20]. Some enzymes involved in FA synthesis, such as cyclooxygenases (COXs), lipoxygenases (LOXs), FASN, acyl-CoA synthetase long-chain family member (ACSL)-1, and peroxisome proliferator-activated receptor gamma (PPARG), alter oxidative stress or antioxidant activity in LPS-treated bovine MECs [21]. This evidence illustrates that enzymes associated with FA metabolism are closely associated with inflammatory diseases caused by abnormal or unbalanced FA metabolism.

Overall, exploring the roles of enzymes associated with FA metabolism and the inflammatory response will provide an innovative direction for understanding physiological and pathological processes in animals, particularly in dairy cows with mastitis. However, in dairy cows, the relationships among FA metabolism, FA-associated enzymes, and CM, in addition to the underlying mechanisms, are not fully understood. The aim of this study was to screen differentially expressed proteins (DEPs) associated with FA metabolism and investigate their functions in healthy dairy cows and those with CM. The expression patterns and distributions of core DEPs were evaluated in the mammary glands of healthy and CM-affected dairy cows, which could provide new strategies for the prevention and treatment of bovine mastitis.

## 2. Results

### 2.1. Identification of Candidate DEPs Related to FA Synthesis or Metabolism Based on GO Terms

**Figure 1:**
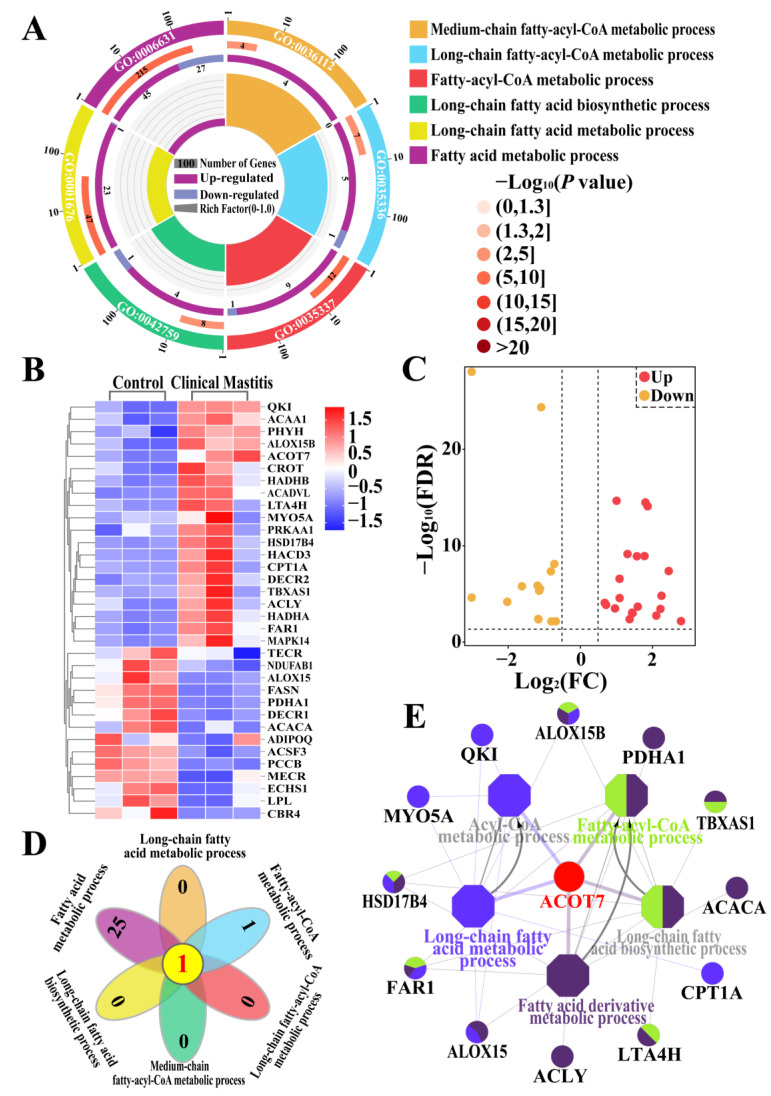
Identification of candidate DEPs and BPs related to FA synthesis or metabolism based on the GO enrichment data. (**A**) Candidate DEPs and BPs related to FA synthesis or metabolism. (**B**) Heat map of 34 candidate DEPs. (**C**) Volcano plot of 34 DEPs, including 14 downregulated and 20 upregulated DEPs. (**D**) Venn diagram of candidate DEPs in six BPs. (**E**) Interactive relationship analysis of candidate DEPs and BPs related to FA synthesis or metabolism using ClueGo.

Gene Ontology (GO) terms related to FA synthesis or metabolism and associated candidate DEPs were screened based on 3739 DEPs and 819 biological process (BP) terms from data-independent acquisition (DIA) proteomics (Figure 1). Six BPs (*p* < 0.05 and *p*.adjust < 0.05; Table S1), including the FA metabolic process, fatty-acyl-CoA metabolic process, LCFA metabolic process and biosynthetic process, and LCFA and MCFA fatty-acyl-CoA metabolic processes, and 34 DEPs (Table S2) were identified (Figure 1A). The heat map and volcano plot showed that these proteins, including 14 DEPs for which expression was downregulated and 20 for which expression was upregulated, were differentially expressed between the Con/C and CM groups (Figure 1B,C). The Venn diagram further indicated that only acyl-CoA thioesterase 7 (ACOT7) was shared among the six BPs (Figure 1D). ClueGO results indicated that 13 of the 34 DEPs were directly related to five BPs, and ACOT7, an important candidate DEP, participates in five BPs related to FA biosynthesis or metabolism.

### 2.2. Identification of Candidate DEPs Related to FA Synthesis or Metabolism in the Pathways

**Figure 2:**
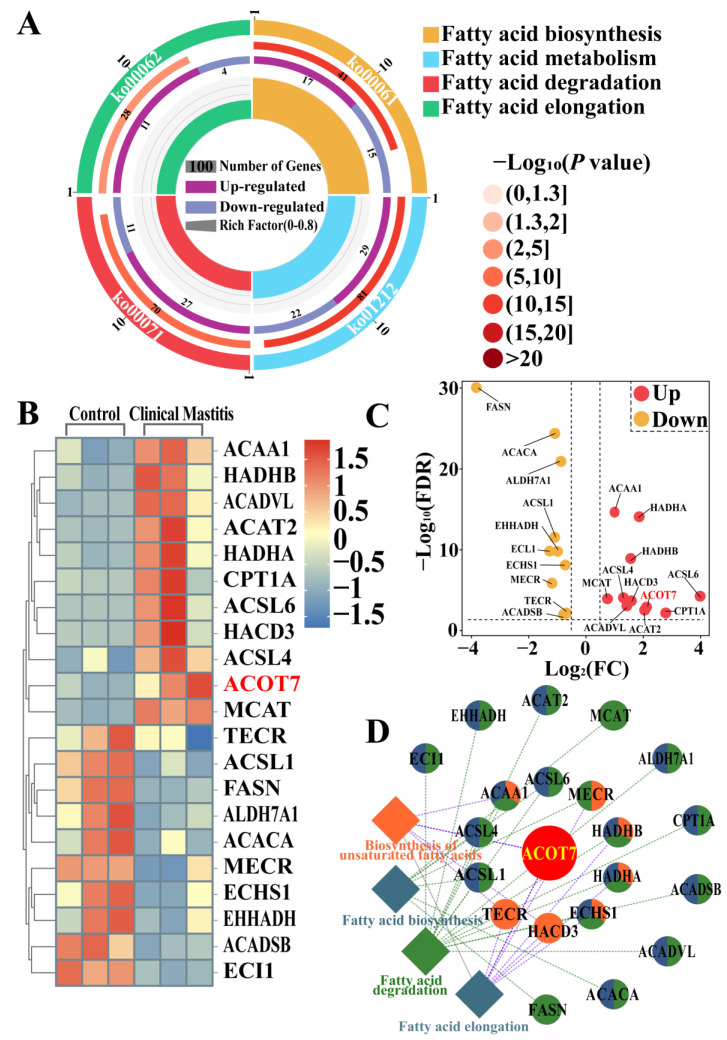
Identification of candidate DEPs and pathways related to FA synthesis or metabolism based on pathway enrichment. (**A**) Candidate DEPs and pathways related to FA synthesis or metabolism. (**B**) Heat map of 21 candidate DEPs. (**C**) Volcano plot of 21 DEPs, including 10 downregulated and 11 upregulated DEPs. (**D**) Interactive relationship analysis of candidate DEPs and pathways related to FA synthesis or metabolism using ClueGo.

Pathways related to FA synthesis or metabolism and their associated DEPs were identified according to a Kyoto Encyclopedia of Genes and Genomes (KEGG) enrichment analysis of the DIA proteomic data (Figure 2). Four pathways (Table S3) fulfilling the *p* < 0.05 and *p*.adjust < 0.05 criteria were obtained (Figure 2A). The heat map and volcano plot results showed that 21 DEPs (Table S4), including 11 for which expression was upregulated and 10 for which expression was downregulated, were screened in the CM group after overlapping them with recurrent DEPs (Figure 2B,C). The protein–protein interaction (PPI) network suggested that four, six, twelve, and seven DEPs directly participate in the four respective pathways (Figure 2D). Additionally, ACOT7 is involved in regulating three identified pathways, namely the biosynthesis of unsaturated fatty acids (UFAs), FA degradation, and FA elongation.

### 2.3. Identification of Core DEPs Related to FA Synthesis or Metabolism Based on Conjoint Analysis of BPs and Pathways

**Figure 3:**
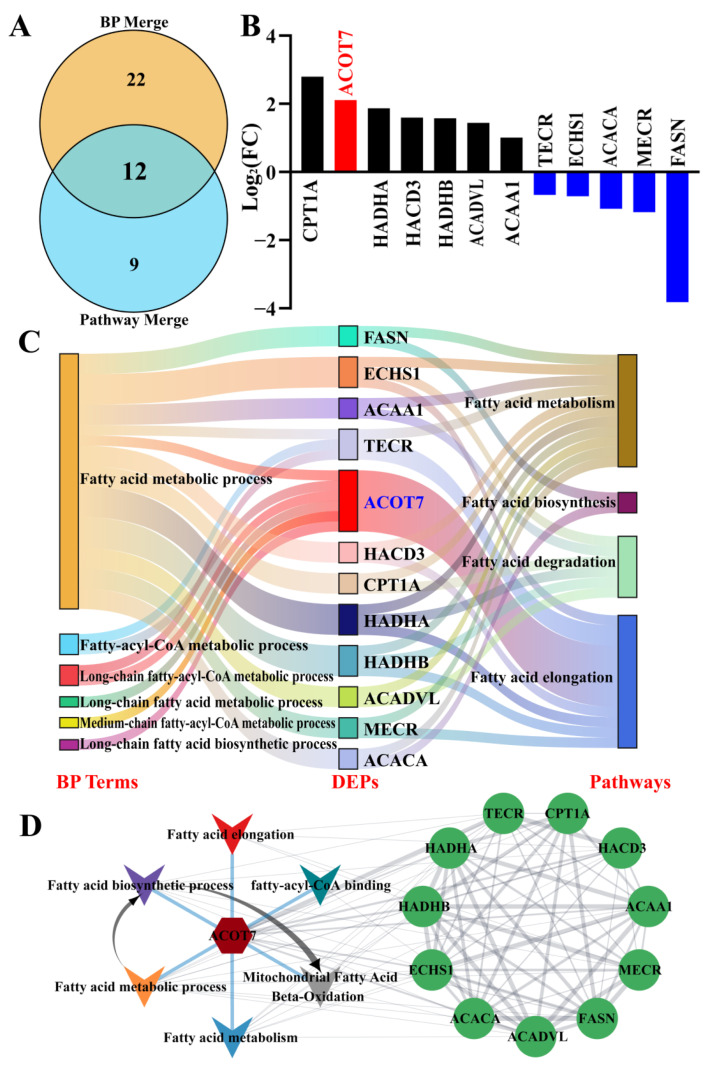
Identification of core DEPs related to FA synthesis or metabolism based on BPs and pathways. (**A**) Venn diagram of candidate DEPs among the merged BPs and pathways. (**B**) Relative expression levels of 12 DEPs quantified by DIA proteomics; the *y*-axis represents the log_2_ (FC) values. (**C**) Sankey diagram of the BPs, shared DEPs, and pathways related to FA synthesis or metabolism. (**D**) Constructed PPI network of the shared DEPs using ClueGO.

The core DEPs related to FA synthesis or metabolism in dairy cows with CM were identified based on the selected BPs and pathways using the conjoint analysis method (Figure 3). A Venn diagram showed that 12 of the 43 candidate DEPs were co-expressed in six BPs and four pathways involved in FA synthesis or metabolism (Figure 3A). Among the twelve shared DEPs, expression levels of seven were upregulated and five were downregulated; in particular, expression levels of carnitine O-palmitoyltransferase 1 (CPT1A), ACOT7, and FASN exhibited changes that exceeded two fold (Figure 3B). The Sankey diagram showed that ACOT7, as a core DEP, was associated with six BPs and the FA elongation pathway (Figure 3C). The PPI network further indicated that ACOT7 was a core DEP that interacts with other candidate DEPs and is involved in FA and mitochondrial energy metabolism (Figure 3D).

### 2.4. Morphological Observation and Distribution Analysis of ACOT7 in Bovine Mammary Tissue

**Figure 4:**
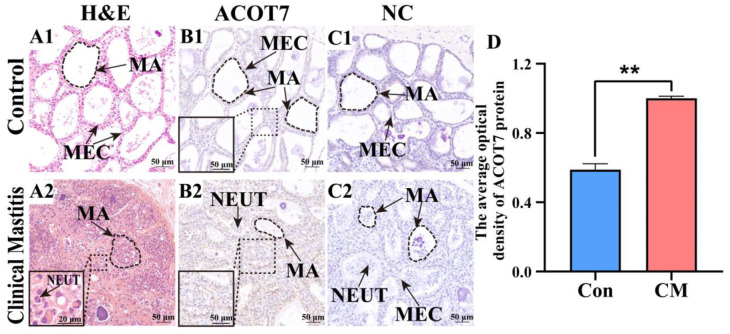
Distribution analysis of ACOT7 in mammary glands of the two groups. (**A**) Pathological variation in bovine mammary glands of the healthy control group (Con/C; **A1**) and clinical mastitis groups (CM; **A2**) based on H&E staining. (**B**) ACOT7 distribution in bovine mammary glands of the Con/C (**B1**) and CM (**B2**) groups based on immunohistochemical staining. (**C**) Negative control (NC) for Con/C (**C1**) and CM (**C2**) groups. (**D**) Gray values of positive expression of ACOT7 protein quantified using ImageJ 1.44p software. Con/C, control group. CM, clinical mastitis group. NC, negative control; MA: mammary alveoli; MECs, mammary epithelial cells; NEUT, neutrophil. Scale bars, 50 μm (200× magnification). ** represents *p* < 0.01.

H&E staining revealed an orderly arrangement of either columnar or cuboidal MECs in the intact alveoli of the bovine mammary gland tissues of the Con group. In contrast, epithelial cells that were detached from mammary tissue, along with predominant neutrophil infiltration in atrophied and deformed alveoli, were observed in the mammary gland tissues of the CM group (Figure 4A). Immunohistochemistry staining (IHC) indicated that ACOT7 was primarily expressed in the cytoplasm of MECs, with increased positive staining observed in the CM group (Figure 4B). No positive ACOT7 staining was observed in the negative control group (Figure 4C). The integrated optical density (IOD) results indicated significantly upregulated expression of ACOT7 in the CM group (*p* < 0.01) compared to that in the Con group (Figure 4D).

### 2.5. Co-Localization Analysis of ACOT7 Protein in Bovine Mammary Tissue

**Figure 5:**
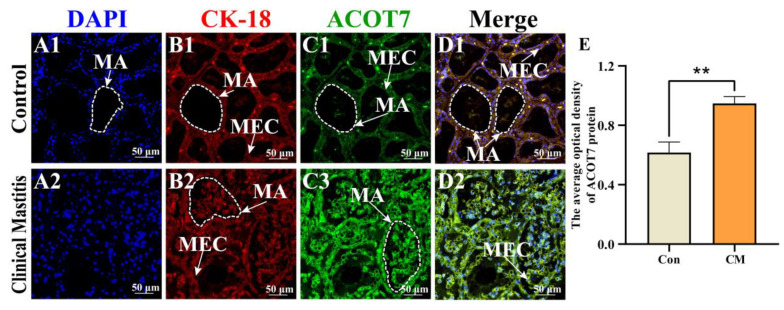
Co-localization analysis of ACOT7 in mammary glands of the two groups. (**A**–**D**) Co-localization analysis of ACOT7 in bovine mammary glands of the Con/C and CM groups: nuclei (blue, **A1**,**A2**), CK-18 (red, **B1**,**B2**), ACOT7 (green, **C1**,**C2**), merged with CK18 and ACOT7 (**D1**,**D2**) staining, respectively. (**E**) Positive IF signals of ACOT7 protein quantified using ImageJ software. Con/C, control group. CM, clinical mastitis group. MA, mammary alveoli; MECs, mammary epithelial cells; Scale bars, 50 μm (200× magnification). ** represents *p* < 0.01.

IF results revealed that nuclei labeled with 4′,6-Diamidino-2-phenylindole (DAPI) appeared in alveoli with an orderly arrangement in the Con group, whereas irregular alveoli were observed in the CM group (Figure 5A). The expression of Cytokeratin (CK)-18 protein, a marker of epithelial cells, showed a similar trend, especially with necrotic and detached MECs present in the incomplete alveoli of the CM group (Figure 5B). Positive fluorescence ACOT7 signals were also present in the MECs of the alveoli in both the Con and CM groups, with different degrees of staining (Figure 5C). The co-localization results suggested that CK-18 and ACOT7 were co-expressed in the cytoplasm of MECs (Figure 5D). The IOD results of IF staining indicated significantly upregulated expression of ACOT7 in the CM group (*p* < 0.01) compared to that in the Con group (Figure 5E). These findings suggest that co-localization of ACOT7 and CK-18 proteins are correlated with the MECs’ function and the occurrence of CM in dairy cows.

### 2.6. ACOT7 mRNA and Protein Expression Patterns in Bovine Mammary Tissue

**Figure 6:**
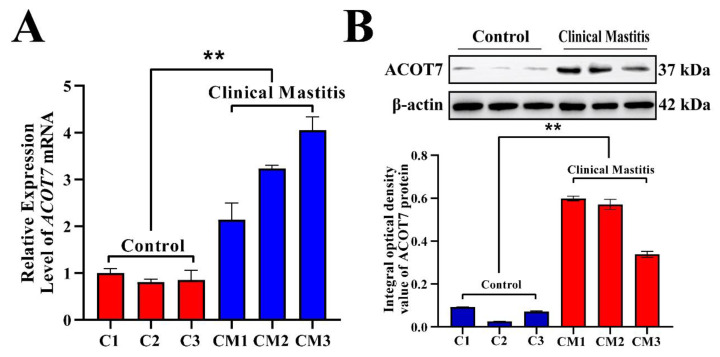
Expression patterns of *ACOT7* mRNA and protein in mammary glands of the two groups. (**A**) Relative expression level of *ACOT7* mRNA, monitored via qRT-PCR assays. (**B**) Protein bands and relative expression level of ACOT7, monitored via Western blot assays and using ImageJ software, respectively. Con/C, control group. CM, clinical mastitis group. Data are presented as means ± SEM. ** represents *p* < 0.01.

The relative expression levels of *ACOT7* mRNA and protein were evaluated in bovine mammary tissues using qRT-PCR and Western blotting (Figure 6). Compared with that in the Con group, the relative expression level of *ACOT7* mRNA was significantly upregulated in the mammary gland tissues of the CM group (Figure 6A). Moreover, ACOT7 protein expression was present but different in each sample of the Con and CM groups (Figure 6B). Results of the average IOD of each band showed that the relative expression level of ACOT7 in the CM group was significantly upregulated compared to that in the Con group (*p* < 0.01). These findings suggest that the upregulation of ACOT7 expression is positively correlated with the occurrence of CM in dairy cows.

## 3. Discussion

Studies have indicated rapid energy metabolism in dairy cows in peak lactation or high-milk-yield stages, which prioritizes the mobilization of body fat [22,23]. The catabolism of FAs (i.e., beta-oxidation) not only provides energy to the body but also generates a series of metabolites with important roles in transcriptional control and cell signaling [24]. Some specific FA metabolites (i.e., SCFAs and n-3 polyunsaturated FAs) have anti-inflammatory effects, whereas others (i.e., LCFAs) promote inflammatory responses [25,26]. The unbalanced production of various FAs or overburdened lipid mobilization in FA metabolism causes a negative energy balance and/or disproportional energy metabolism. These metabolic disturbances impair immune function, reduce fertility and milk production, and ultimately decrease the economic benefits associated with dairy cows affected by mastitis [9]. Enzymes associated with FA metabolism and their metabolites are closely associated with FA lipolysis and synthesis in cow mastitis [2,4]. This study offers novel insights by identifying ACOT7 as a key enzyme that connects fatty acid metabolism to inflammation, thereby addressing a significant gap in the literature concerning its role in dairy cows with mastitis. However, the systematic exploration of using enzymes or proteins to elucidate this relationship requires further refinement.

In the present study, candidate DEPs related to FA synthesis or metabolism were screened based on GO terms and pathways in the DIA proteomic data generated from the mammary gland tissues of lactating Holstein cows with or without CM. Six BPs involving 34 DEPs and four pathways involving 21 DEPs related to FA synthesis and metabolism were identified. Among the DEPs, eight were enriched in three long-chain FA metabolic processes, and seven of the eight were involved in the FA metabolic process, especially ACOT7, indicating that LCFA metabolic processes are associated with the onset and progression of CM in dairy cows. LCFAs cause significant changes in the blood and milk samples of cows with CM, which could be related to the inflammatory response, metabolic status, and milk fat composition [27,28]. LCFAs are organic compounds that support various biological processes and maintain homeostasis [29]. For example, lauric acid, palmitic acid, and stearic acid are LCFAs that can promote persistent inflammation and induce Cyclooxygenase-2 (COX-2) expression through a nuclear factor kappa-B (NF-κB)-dependent mechanism in macrophages [14]. Further, SFAs are crucial components of bacterial endotoxins and can indirectly stimulate TLR-dependent signaling [30,31,32], which is also the main mechanism through which pathogenic bacteria infect the mammary tissue during the occurrence of cow mastitis. In addition, the identified pathways, including FA biosynthesis, metabolism, degradation, and elongation, are indispensable for FA metabolism. Previous studies have suggested that FASN in FA biosynthesis [33], CPT1A in FA metabolism [34], ACSLs in FA degradation [35], and very-long-chain enoyl-CoA reductase (TECR) in FA elongation are associated with the development of cow mastitis [36]. A further bioinformatic analysis indicated that LCFA metabolic pathways have a direct relationship with ACOT7 expression, suggesting that ACOT7 might influence the development of CM through its role in FA synthesis or metabolism.

ACOT7, a predominant member of the ACOT family, is an extensively studied protein that regulates the intracellular levels of FAs, acyl-CoA, and coenzyme A by catalyzing the hydrolysis of acyl-CoA to produce free fatty acids (FFAs) and coenzyme A [18]. By regulating the cell cycle, ACOT7 drives the development of mammary gland diseases; moreover, ACOT7 specifically hydrolyzes arachidonoyl-CoA to release arachidonic acid [18,19], underscoring its close association with LCFAs. In mouse macrophages, LPS-induced *ACOT7* mRNA expression is mediated by the TLR4-Myeloid Differentiation Primary Response 88 (MyD88) pathway, underscoring its role in inflammation. Similarly, ACOT7 plays a critical role in neurotoxicity prevention by modulating neuronal fatty acid metabolism, thus providing protection to the nervous system [37]. These studies highlight the broad physiological functions of the ACOT7 family in fatty acid metabolism and inflammation, suggesting their potential involvement in various inflammatory conditions. These findings bear similarities to our results; however, reports on the involvement of ACOT7 in CM development through fatty acid pathways in Holstein cows are limited. In this study, DIA proteomic results indicated high ACOT7 expression in the CM group and suggested that it participates in the regulation of FA elongation and UFA biosynthesis, which is consistent with the mRNA and protein expression findings. The elevated expression of ACOT7 may represent an adaptive response in dairy cows during lactation, a period of negative energy balance, where it increases the release of free fatty acids for β-oxidation to meet the energy demands. The biosynthesis of UFAs is closely related to LCFA elongation. In addition, these processes function together in multiple organisms to produce FAs of varying lengths and saturation levels, such as polyunsaturated fatty acids [38], which are involved in the regulation of inflammatory responses [27] and antibacterial activity [39], and influence gene expression and the gut microbiota [40] in dairy cows with mastitis. Co-localization results also confirmed that ACOT7 was predominantly expressed in the cytoplasm of MECs. These findings suggest that high ACOT7 expression in CM affects FA metabolism, leading to the decomposition of long-chain acyl-CoA and the release of FFAs that stimulate TLR4 receptor signaling, thereby influencing the onset of CM in dairy cows [14].

**Figure 7:**
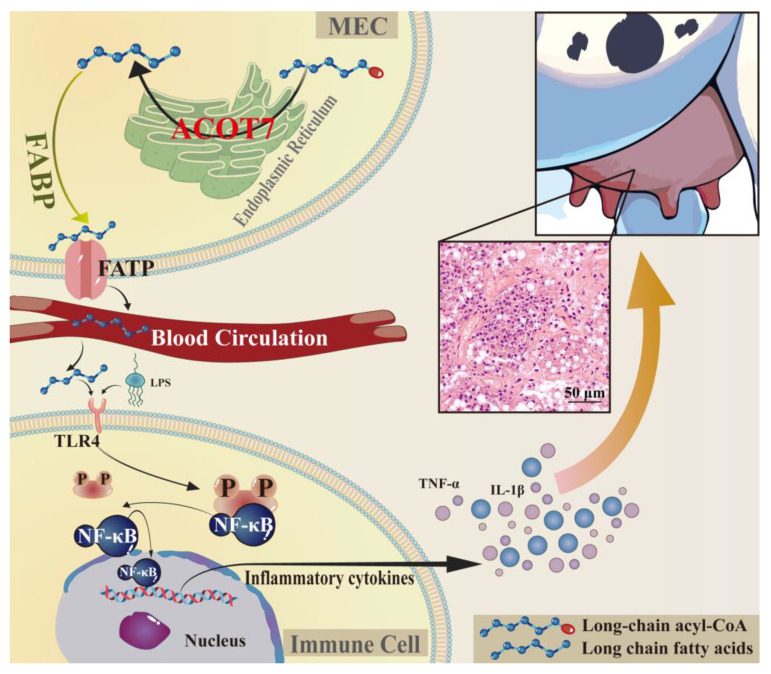
Proposed molecular mechanism underlying the effects of ACOT7 in the mammary glands of dairy cows with clinical mastitis (CM). MECs, mammary epithelial cells; FABP, fatty-acid-binding protein; FATP, fatty acid transport protein; TLR4, Toll-like receptor 4; TNF-α, tumor necrosis factor-α; IL-1β, interleukin-1β; LPS, lipopolysaccharide. Scale bar, 50 μm (200× magnification).

Based on these experimental results and previous research, we propose a potential mechanism underlying the role of ACOT7 in CM (Figure 7). Specifically, ACOT7 hydrolyzes long-chain acyl-CoA to acetyl-CoA in the endoplasmic reticulum, thereby promoting LCFA synthesis [41]. These FAs are transported to the extracellular space and bloodstream by FA transport proteins (FATPs) and FA-binding proteins (FABPs) [42,43]. This process activates the TLR4 pathway, triggers NF-κB signaling cascades, and ultimately promotes the expression and release of inflammatory factors, such as TNF-α and IL-1β, thereby enhancing the inflammatory response. However, this study still has certain limitations, and validated assays based on results at the cellular and animal level need to confirm this. Addressing these aspects would enhance the credibility of the interpretations. Nevertheless, we systematically identified candidate DEPs related to FA synthesis or metabolism that contribute to the onset and progression of CM in dairy cows, providing new insights into the prevention and treatment of mastitis in dairy cows. In addition to the fundamental findings on the role of ACOT7 and fatty acid metabolism in CM, this study holds significant practical implications for dairy farming. Moreover, the findings on ACOT7 provide a theoretical basis for the identification of new drug targets and potential biomarkers for CM.

## 4. Materials and Methods

### 4.1. Tissue Sample Preparation

The lactating Holstein cows utilized in the present study were randomly selected from a commercially managed dairy farm in Wuzhong City (Ningxia Hui Autonomous Region, China). Veterinary clinical diagnoses and milk tests, including somatic cell count (SCC) and the Lanzhou Mastitis Test (LMT), were performed as previously described [44,45]. Holstein cows without typical clinical changes, with an SCC of 7 × 10^4^–1 × 10^5^ cells/mL and a negative LMT, were classified as the 3 healthy control group (Con/C). In contrast, Holstein cows with typical disease signs, an SCC of 1 × 10^5^–15 × 10^5^ cells/mL, and a positive LMT were assigned to the CM group. To ensure similarity in age, parity, body condition (aged 5–7 years, 3rd to 5th lactation, 60–250 days in milk), and the absence of other diseases, cows in the Con/C and CM groups (*n* = 3 per group) were transported to the slaughterhouse, where mammary gland samples were collected after slaughter. The tissues were immediately fixed in 4% paraformaldehyde, or promptly frozen in liquid nitrogen, and stored at −80 °C in the laboratory. This study was approved by the Ethics Committee of Gansu Agricultural University (No. GSAU-AEW-2018-0128).

### 4.2. Bioinformatic Analysis

GO and KEGG annotations were performed based on DIA proteomic sequencing data from mammary gland samples from the Con/C and CM groups of Holstein cows (accession number: IPX0003382000/PXD028100). A bioinformatic analysis was conducted to identify GO categories, KEGG pathways, and DEPs related to FA synthesis or metabolism, with significance set at *p* < 0.05 and *p*.adjust < 0.05. Venn diagrams, enrichment circle plots, heat maps, and volcano plots were created using R language and the OmicShare online platform (http://www.omicshare.com/tools, accessed on 20 February 2024) [45]. PPI networks were constructed to screen the potential candidate functional DEPs related to FA synthesis or metabolism using STRING v12.0 (EMBL, Heidelberg, Germany), Cytoscape 3.10.0 (Cytoscape Consortium, La Jolla, CA, USA), and ClueGO 2.5.10 software (Cytoscape Consortium, La Jolla, CA, USA). The potential mechanisms associated with the candidate DEPs were predicted through KEGG analysis and a literature review, and mechanistic diagrams were created using Adobe Illustrator 2022 (Adobe, San Jose, MA, USA) and MedPeer 2.4.241127 (MedPeer^®^, Beijing, China).

### 4.3. Histochemistry Staining

Fixed mammary gland tissues were processed using standard paraffin (Solarbio, Beijing, China)-embedding techniques and sectioned into 5 μm thick slices using a microtome (Leica, Wetzlar, Germany), as described previously [45]. H&E staining was performed as previously described [45,46]. Briefly, after deparaffinization in xylene and dehydration in a gradient of ethanol, the slices were stained using an H&E staining solution kit (Servicebio, Wuhan, China). The slices were examined and imaged using an optical microscope and imaging system (Nikon, Tokyo, Japan). Six fields from each view were randomly selected for each sample.

### 4.4. Immunohistochemistry Staining (IHC)

After deparaffinization and hydration, slices were subjected to antigen retrieval using sodium citrate (Servicebio, Wuhan, China). Endogenous catalase was deactivated and blocked using 3% H_2_O_2_ and 10% donkey serum, respectively (Solarbio, Beijing, China). The slices were incubated with a rabbit anti-ACOT7 primary antibody at a 1:200 dilution (Proteintech, Wuhan, China). Secondary antibody incubation and chromogenic reactions were performed using an StreptAvidin–Biotin Complex (SABC) staining kit (Boster, Wuhan, China) and a 3-diaminobenzidine (DAB) substrate kit (Solarbio, Beijing, China), as described previously [46]. Phosphate-Buffered Saline (PBS) was used instead of primary antibodies in the NC group. The sections were examined and imaged using a Zeiss LSM 800 confocal microscope (Carl Zeiss, Oberkochen, Germany). Ten random fields of view were selected for each section. ImageJ 1.44p software (National Institutes of Health, Rockville, MD, USA) was used to measure the average positive expression in each field, and the relative expression levels were analyzed. All immunostaining assays and measurements were performed in triplicate.

### 4.5. Immunofluorescence Staining (IF)

The blocked slices were incubated with rabbit anti-CK-18 and rabbit anti-ACOT7 primary antibodies (1:100; Proteintech, Wuhan, China), as described previously [46]. The slices were incubated with fluorescent secondary antibodies with different labels (1:350 dilution; Alexa Fluor^®^ 488 for ACOT7 and Alexa Fluor^®^ 647 for CK-18, Proteintech, Wuhan, China), as described previously [46]. The nuclei were stained with 0.5 µg/mL DAPI (Solarbio, Beijing, China). Finally, the sections were observed and imaged using an Echo-Labs upright/inverted fluorescence microscope and imaging system (Olympus, Tokyo, Japan). The positive IF signals were measured using ImageJ software, as described in IHC staining. All staining assays and measurements were performed in triplicate.

### 4.6. RNA Extraction, cDNA Synthesis, and qRT-PCR Detection

Total RNA was extracted from the tissues (20 mg of each tissue sample frozen at −80 °C) of the Con/C and CM groups using the FastPure RNA isolation kit (Vazyme, Nanjing, China), RNA quantification was performed using a NanoDrop-8000 spectrophotometer (Thermo Fisher Scientific, Waltham, MA, USA), and RNA integrity was evaluated through 1% denaturing formaldehyde agarose gel electrophoresis. And, a 500 ng RNA sample was used for complementary DNA (cDNA) synthesis using the BioTeke Thermo RT Kit (BioTeke, Beijing, China) according to the manufacturer’s instructions, as previously described [45,47]. Primers for bovine β-actin and ACOT7 (Table S5) were designed using Primer Premier 5.0 software (PREMIER Biosoft, San Francisco, CA, USA) and synthesized by Qingke Biological Technology Co., Ltd. (Shanxi, China). A qRT-PCR was performed using cDNA as the template, as previously described [46]. The relative expression levels of β-actin were taken as the internal control. The results were calculated using the 2^−∆∆Ct^ method. All qRT-PCR assays were performed at least in triplicate.

### 4.7. Western Blotting

Total protein was isolated from the tissues (100 mg per sample stored at −80 °C) of the Con and CM groups using a Radioimmunoprecipitation Assay (RIPA) kit (Solarbio, Beijing, China) and quantified using a Bicinchoninic Acid Protein Assay (BCA) kit (Solarbio), according to the manufacturer’s instructions. Total proteins (50 μg) were used to detect the expression levels of ACOT7 with a 1:800 dilution ratio, and β-actin with a 1:4500 dilution ratio (Bioss, Beijing, China), in the Con/C and CM groups, as described previously [45,47]. The bands were visualized using Enhanced Chemiluminescence (ECL) (Solarbio, Beijing, China) and analyzed using ImageJ. All immunoblot assays were performed at least in triplicate.

### 4.8. Statistical Analysis

Statistical analysis was performed using SPSS 23.0 (SPSS Inc., Chicago, IL, USA). Data are expressed as means ± standard deviations (X ± SD), unless otherwise stated. Comparisons between groups were performed using the least significant difference test, whereas multiple group comparisons were performed via one-way ANOVA. Statistical significance was set at *p* < 0.05. Graphs were created using GraphPad Prism 8.0.2 (GraphPad Software Inc., San Diego, CA, USA).

## 5. Conclusions

Based on DIA proteomics data, we identified 34 DEPs associated with six BPs, and four pathways involving 21 DEPs related to fatty acid metabolism and synthesis. Some DEPs such as CPT1A, FASN, and TECR regulate fatty acid metabolism and CM development by modulating pathways such as fatty acid β-oxidation, the fatty acid metabolic process, and the long-chain fatty-acyl-CoA metabolic process. Crucially, comprehensive analysis indicates that ACOT7 functions as a key enzyme potentially involved in the pathogenesis of CM in dairy cows via fatty acid metabolic pathways. Furthermore, IHC and IF staining demonstrated that ACOT7 was primarily localized in the MECs of dairy cows. Notably, compared to the Con/C group, the *ACOT7* mRNA and protein levels were significantly upregulated in the mammary glands of the CM group. Therefore, this study proposes a mechanism of ACOT7, which may contribute to the development of CM. Combining proteomics could help define the function of ACOT7 in CM, shedding light on its potential as a predictive biomarker or even a therapeutic target for novel CM treatment strategies using knockout models, and providing a theoretical foundation for further investigation into the physiological roles of fatty acids in the mammary tissues of dairy cows.

## Data Availability

The data that support the findings of this study are available from the corresponding author upon reasonable request.

## Supplementary Materials

The following supporting information can be downloaded at: https://www.mdpi.com/article/10.3390/ijms252313046/s1.

## References

[1] Sordillo L.M., Contreras G.A., Aitken S.L. (2009). Metabolic Factors Affecting the Inflammatory Response of Periparturient Dairy Cows. Anim. Health Res. Rev..

[2] Zhang H., Shen Z., Yang Z., Jiang H., Chu S., Mao Y., Li M., Chen Z., Aboragah A., Loor J.J. (2021). Abundance of Solute Carrier Family 27 Member 6 (*SLC27A6*) in the Bovine Mammary Gland Alters Fatty Acid Metabolism. Food Funct..

[3] Marshall J.C. (2005). Lipopolysaccharide: An Endotoxin or an Exogenous Hormone?. Clin. Infect. Dis..

[4] Luo Y., Kong Z., Yang B., He F., Huan C., Li J., Yi K. (2023). Relationship between Microflora Changes and Mammary Lipid Metabolism in Dairy Cows with Mastitis. Animals.

[5] Wang Y., Nan X., Zhao Y., Jiang L., Wang H., Zhang F., Hua D., Liu J., Yang L., Yao J. (2022). Discrepancies among Healthy, Subclinical Mastitic, and Clinical Mastitic Cows in Fecal Microbiome and Metabolome and Serum Metabolome. J. Dairy Sci..

[6] Calder P.C. (2015). Marine Omega-3 Fatty Acids and Inflammatory Processes: Effects, Mechanisms and Clinical Relevance. Biochim. Biophys. Acta (BBA) Mol. Cell Biol. Lipids.

[7] Li L., Tang W., Zhao M., Gong B., Cao M., Li J. (2021). Study on the Regulation Mechanism of Lipopolysaccharide on Oxidative Stress and Lipid Metabolism of Bovine Mammary Epithelial Cells. Physiol. Res..

[8] Calder P.C. (2012). Long-Chain Fatty Acids and Inflammation. Proc. Nutr. Soc..

[9] Esposito G., Irons P.C., Webb E.C., Chapwanya A. (2014). Interactions between Negative Energy Balance, Metabolic Diseases, Uterine Health and Immune Response in Transition Dairy Cows. Anim. Reprod. Sci..

[10] Vinolo M.A.R., Rodrigues H.G., Nachbar R.T., Curi R. (2011). Regulation of Inflammation by Short Chain Fatty Acids. Nutrients.

[11] Akhtar M., Naqvi S.U.-A.-S., Liu Q., Pan H., Ma Z., Kong N., Chen Y., Shi D., Kulyar M.F.-E.-A., Khan J.A. (2022). Short Chain Fatty Acids (SCFAs) Are the Potential Immunomodulatory Metabolites in Controlling *Staphylococcus aureus*-Mediated Mastitis. Nutrients.

[12] Chait A., Kim F. (2010). Saturated Fatty Acids and Inflammation: Who Pays the Toll?. Arterioscler. Thromb. Vasc. Biol..

[13] Rocha D.M., Caldas A.P., Oliveira L.L., Bressan J., Hermsdorff H.H. (2016). Saturated Fatty Acids Trigger TLR4-Mediated Inflammatory Response. Atherosclerosis.

[14] Rogero M., Calder P. (2018). Obesity, Inflammation, Toll-Like Receptor 4 and Fatty Acids. Nutrients.

[15] Aitken S.L., Corl C.M., Sordillo L.M. (2011). Immunopathology of Mastitis: Insights into Disease Recognition and Resolution. J. Mammary Gland. Biol. Neoplasia.

[16] Ingvartsen K.L., Moyes K. (2013). Nutrition, Immune Function and Health of Dairy Cattle. Animal.

[17] Wang Z., Wang Q., Tang C., Yuan J., Luo C., Li D., Xie T., Sun X., Zhang Y., Yang Z. (2023). Medium Chain Fatty Acid Supplementation Improves Animal Metabolic and Immune Status during the Transition Period: A Study on Dairy Cattle. Front. Immunol..

[18] Forwood J.K., Thakur A.S., Guncar G., Marfori M., Mouradov D., Meng W., Robinson J., Huber T., Kellie S., Martin J.L. (2007). Structural Basis for Recruitment of Tandem Hotdog Domains in Acyl-CoA Thioesterase 7 and Its Role in Inflammation. Proc. Natl. Acad. Sci. USA.

[19] Jung S.H., Lee H.C., Hwang H.J., Park H.A., Moon Y.-A., Kim B.C., Lee H.M., Kim K.P., Kim Y.-N., Lee B.L. (2017). Acyl-CoA Thioesterase 7 Is Involved in Cell Cycle Progression via Regulation of PKCζ–P53–P21 Signaling Pathway. Cell Death Dis..

[20] Wall V.Z., Barnhart S., Kramer F., Kanter J.E., Vivekanandan-Giri A., Pennathur S., Bolego C., Ellis J.M., Gijón M.A., Wolfgang M.J. (2017). Inflammatory Stimuli Induce Acyl-CoA Thioesterase 7 and Remodeling of Phospholipids Containing Unsaturated Long (≥C20)-Acyl Chains in Macrophages [S]. J. Lipid Res..

[21] Ma N., Wei G., Zhang H., Dai H., Roy A.C., Shi X., Chang G., Shen X. (2021). Cis-9, Trans-11 CLA Alleviates Lipopolysaccharide-Induced Depression of Fatty Acid Synthesis by Inhibiting Oxidative Stress and Autophagy in Bovine Mammary Epithelial Cells. Antioxidants.

[22] Kuhla B., Metges C.C., Oltjen J.W., Kebreab E., Lapierre H. (2013). Proteomic Tools Help Understanding the Metabolic Adaptation to Negative Energy Balance in Dairy Cows. Energy and Protein Metabolism and Nutrition in Sustainable Animal Production, Proceedings of the 4th International Symposium on Energy and Protein Metabolism and Nutrition, Sacramento, CA, USA, 9–12 September 2013.

[23] Abiso M., Yohannes B., Alemu B. (2019). Combating Negative Effect of Negative Energy Balance in Dairy Cows: Comprehensive Review. Approaches Poult. Dairy Vet. Sci..

[24] Wang Z., Hou X., Shang G., Deng G., Luo K., Peng M. (2024). Exploring Fatty Acid β-Oxidation Pathways in Bacteria: From General Mechanisms to DSF Signaling and Pathogenicity in Xanthomonas. Curr. Microbiol..

[25] Corrêa R.O., Vieira A., Sernaglia E.M., Lancellotti M., Vieira A.T., Avila-Campos M.J., Rodrigues H.G., Vinolo M.A.R. (2017). Bacterial Short-Chain Fatty Acid Metabolites Modulate the Inflammatory Response against Infectious Bacteria. Cell. Microbiol..

[26] Jeong H.Y., Moon Y.S., Cho K.K. (2024). ω-6 and ω-3 Polyunsaturated Fatty Acids: Inflammation, Obesity and Foods of Animal Resources. Food Sci. Anim. Resour..

[27] Mavangira V., Gandy J.C., Zhang C., Ryman V.E., Jones A.D., Sordillo L.M. (2015). Polyunsaturated Fatty Acids Influence Differential Biosynthesis of Oxylipids and Other Lipid Mediators during Bovine Coliform Mastitis. J. Dairy Sci..

[28] Atroshi F., Rizzo A., Kangasniemi R., Sankari S., Työppönen T., Österman T., Parantainen J. (1989). Role of Plasma Fatty Acids, Prostaglandins and Antioxidant Balance in Bovine Mastitis. J. Vet. Med. Ser. A.

[29] Jannas-Vela S., Espinosa A., Candia A.A., Flores-Opazo M., Peñailillo L., Valenzuela R. (2023). The Role of Omega-3 Polyunsaturated Fatty Acids and Their Lipid Mediators on Skeletal Muscle Regeneration: A Narrative Review. Nutrients.

[30] Erridge C., Samani N.J. (2009). Saturated Fatty Acids Do Not Directly Stimulate Toll-Like Receptor Signaling. Arterioscler. Thromb. Vasc. Biol..

[31] Anderson E.K., Hill A.A., Hasty A.H. (2012). Stearic Acid Accumulation in Macrophages Induces Toll-like Receptor 4/2-Independent Inflammation Leading to Endoplasmic Reticulum Stress-Mediated Apoptosis. Arterioscler. Thromb. Vasc. Biol..

[32] Lancaster G.I., Langley K.G., Berglund N.A., Kammoun H.L., Reibe S., Estevez E., Weir J., Mellett N.A., Pernes G., Conway J.R.W. (2018). Evidence That TLR4 Is Not a Receptor for Saturated Fatty Acids but Mediates Lipid-Induced Inflammation by Reprogramming Macrophage Metabolism. Cell Metab..

[33] Huma Z.I., Sharma N., Kour S., Tandon S., Guttula P.K., Kour S., Singh A.K., Singh R., Gupta M.K. (2020). Putative Biomarkers for Early Detection of Mastitis in Cattle. Anim. Prod. Sci..

[34] Ceciliani F., Lecchi C., Urh C., Sauerwein H. (2018). Proteomics and Metabolomics Characterizing the Pathophysiology of Adaptive Reactions to the Metabolic Challenges during the Transition from Late Pregnancy to Early Lactation in Dairy Cows. J. Proteom..

[35] Delosière M., Bernard L., Viala D., Fougère H., Bonnet M. (2023). Milk and Plasma Proteomes from Cows Facing Diet-Induced Milk Fat Depression Are Related to Immunity, Lipid Metabolism and Inflammation. Animal.

[36] Cecchinato A., Macciotta N.P.P., Mele M., Tagliapietra F., Schiavon S., Bittante G., Pegolo S. (2019). Genetic and Genomic Analyses of Latent Variables Related to the Milk Fatty Acid Profile, Milk Composition, and Udder Health in Dairy Cattle. J. Dairy Sci..

[37] Xie X., Chen C., Feng S., Zuo S., Zhao X., Li H. (2021). Acyl-CoA Thioesterase 7 Is Transcriptionally Activated by Krüppel-Like Factor 13 and Promotes the Progression of Hepatocellular Carcinoma. J. Hepatocell. Carcinoma.

[38] Cook H.W., Vance D.E., Vance J.E. (1996). Chapter 5—Fatty Acid Desaturation and Chain Elongation in Eukaryotes. New Comprehensive Biochemistry.

[39] Hogan J.S., Smith K.L., Todhunter D.A., Schoenberger P.S. (1988). Growth Responses of Environmental Mastitis Pathogens to Long-Chain Fatty Acids1. J. Dairy Sci..

[40] Feng J., Wang Q., Yang W., Liu J., Gao M.-Q. (2021). Omega-3 Polyunsaturated Fatty Acids Ameliorated Inflammatory Response of Mammary Epithelial Cells and Mammary Gland Induced by Lipopolysaccharide. Acta Biochim. Biophys. Sin..

[41] Yamada J., Kurata A., Hirata M., Taniguchi T., Takama H., Furihata T., Shiratori K., Iida N., Takagi-Sakuma M., Watanabe T. (1999). Purification, Molecular Cloning, and Genomic Organization of Human Brain Long-Chain Acyl-CoA Hydrolase. J. Biochem..

[42] Lipke K., Kubis-Kubiak A., Piwowar A. (2022). Molecular Mechanism of Lipotoxicity as an Interesting Aspect in the Development of Pathological States-Current View of Knowledge. Cells.

[43] Stahl A. (2001). Fatty Acid Transport Proteins: A Current View of a Growing Family. Trends Endocrinol. Metab..

[44] Zhang Q., Bai X., Shi J., Wang X., Zhang B., Dai L., Lin T., Gao Y., Zhang Y., Zhao X. (2022). DIA Proteomics Identified the Potential Targets Associated with Angiogenesis in the Mammary Glands of Dairy Cows with Hemorrhagic Mastitis. Front. Vet. Sci..

[45] Zhang Q., Bai X., Lin T., Wang X., Zhang B., Dai L., Shi J., Zhang Y., Zhao X. (2022). HMOX1 Promotes Ferroptosis in Mammary Epithelial Cells via FTH1 and Is Involved in the Development of Clinical Mastitis in Dairy Cows. Antioxidants.

[46] Zhang Q., Wang Q., Zhang Y., Cheng S., Hu J., Ma Y., Zhao X. (2018). Comprehensive Analysis of MicroRNA^−^Messenger RNA from White Yak Testis Reveals the Differentially Expressed Molecules Involved in Development and Reproduction. Int. J. Mol. Sci..

[47] Zhang B., Lin T., Bai X., An X., Dai L., Shi J., Zhang Y., Zhao X., Zhang Q. (2022). Sulfur Amino Acid Metabolism and the Role of Endogenous Cystathionine-γ-Lyase/H2S in Holstein Cows with Clinical Mastitis. Animals.

