# Supplementary figures and images for: Role of Acyl-CoA Thioesterase 7 in Regulating Fatty Acid Metabolism and Its Contribution to the Onset and Progression of Bovine Clinical Mastitis
Source: Int J Mol Sci. 2024 Dec 4;25(23):13046. doi: 10.3390/ijms252313046 (PMC11642332; doi:10.3390/ijms252313046)

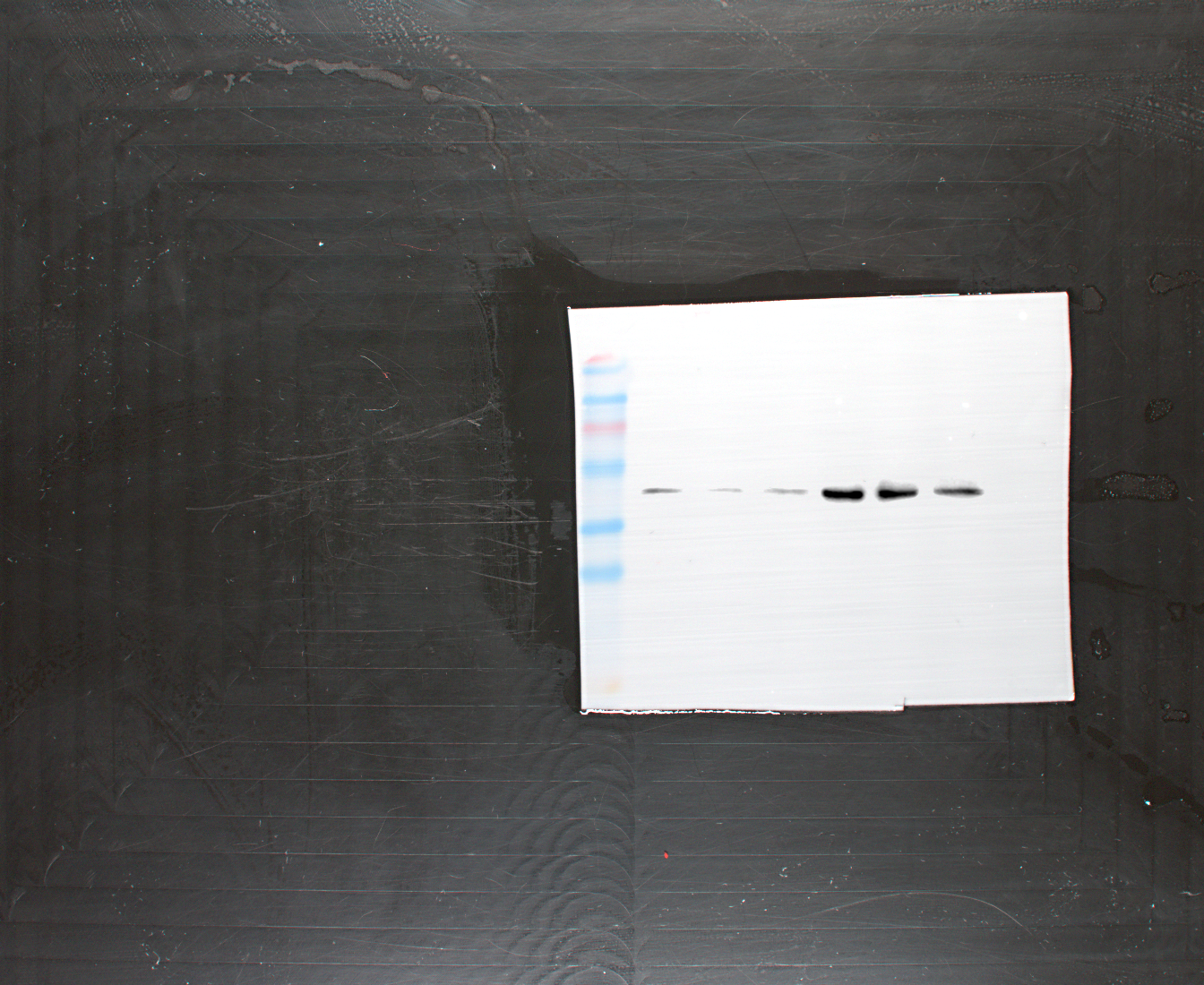


**Figure S1:** Whole Western blot of ACOT7 (37 kDa).


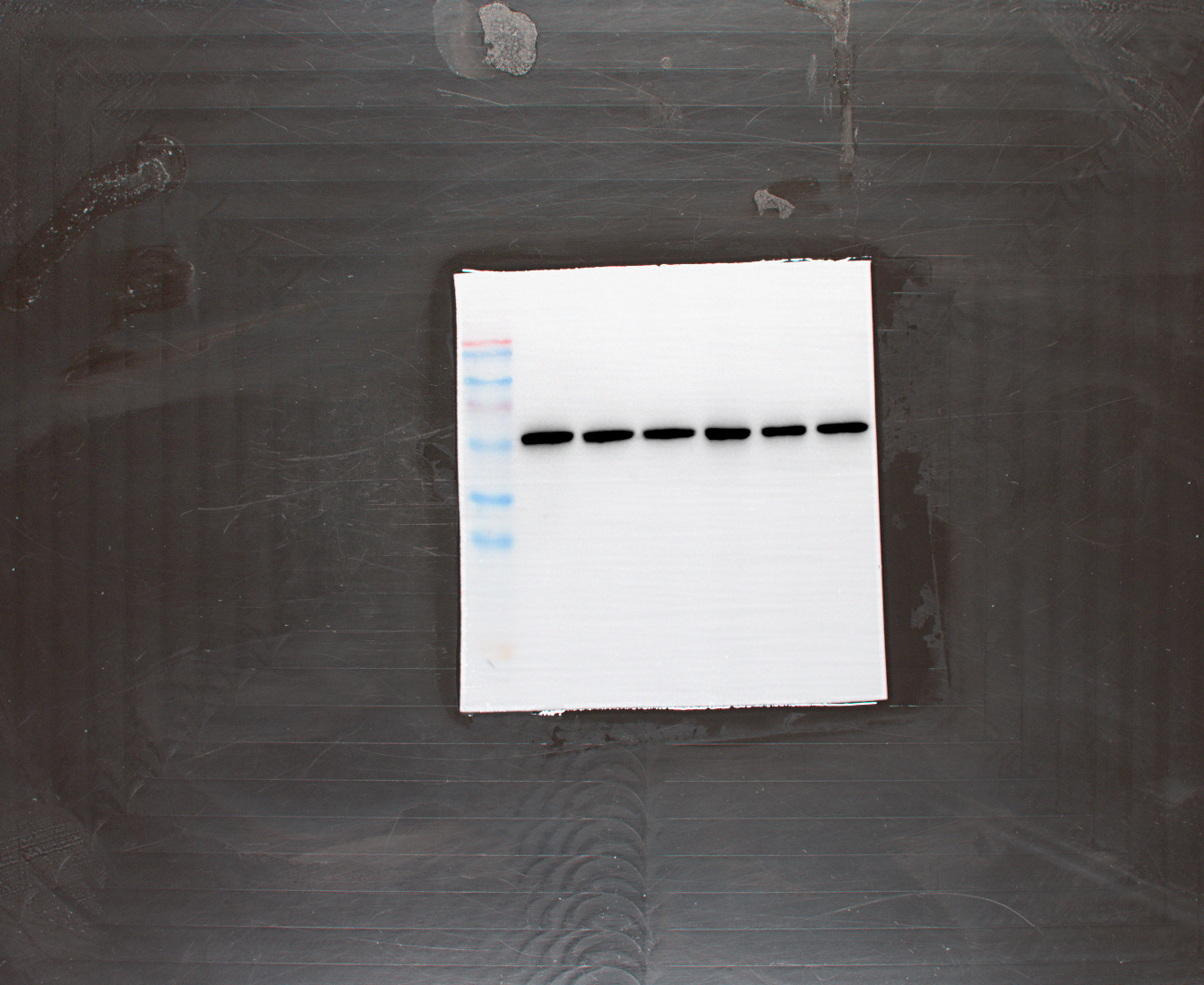


**Figure S2:** Whole Western blot of β-actin (42 kDa).

Supplement: Supplementary file 1 [file ijms-25-13046-s001.zip › Supplementary Figures.docx]
